# Single center experience on implementation of the postpartum intrauterine device (PPIUD) in Sri Lanka: a retrospective study

**DOI:** 10.1186/s13104-020-05045-x

**Published:** 2020-04-10

**Authors:** D. L. W. Dasanayake, M. Patabendige, Y. Amarasinghe

**Affiliations:** 1 Senior Lecturer in Obstetrics and Gynaecology, Faculty of Medicine, University of Ruhuna, Galle, Sri Lanka; 2 Senior Registrar in Obstetrics and Gynaecology, Castle Street Hospital for Women, Ward: 07/08, Colombo, Sri Lanka; 3 Scientific Assistant, Department of Obstetrics and Gynaecology, Faculty of Medicine, University of Ruhuna, Galle, Sri Lanka

**Keywords:** Postpartum period, Family planning services, Intrauterine devices, Copper, Health plan implementation

## Abstract

**Objectives:**

to study the institutionalization of PPIUD services at Teaching Hospital, Mahamodara, Galle (THMG) and to study the feasibility, challenges and barriers for implementation.

**Results:**

Total of 46,815 deliveries had occurred in the facility during the study period. Out of that 23,117 (49.4%) women had been counseled and 5775 (25.0%) of them were primigravida and 892 (3.9%) were teenage mothers. Total of 14,051 (60.8%) women were interviewed, but only 772 (5.5%) women consented for PPIUD. Consent withdrawal was seen in 29 (3.8%) cases. A total of 409 community health staff were trained for counseling and follow up. PPIUD uptake was 470 (3.4%) which is comparable to national figures. Follow up data at 1 year was available from 199 women and spontaneous expulsion had occurred in eight (6.7%) cases with no cases of perforation or failure in terms of pregnancy. This short report gives the impression that PPIUD can be successfully implemented in resource limited settings and this also provides a feedback for the policy makers to take the necessary actions to improve the uptake of this cost effective, safe PPFP method. A routine PPIUD service has been successfully established within a tertiary care maternity setting in Sri Lanka.

## Introduction

Family planning can prevent more than 30% of maternal mortality and 10% of child deaths if couples space their pregnancies more than 2 years apart [1].

## Main text

### Maternity care in Sri Lanka

Notwithstanding the long-overdue success story, Sri Lanka has a stagnation of MMR for more than a decade [2]. As well as unplanned pregnancies resulting in unsafe abortions, short birth interval, low birth weight and increased maternal morbidity and mortality has become a challenging issue towards breaking down of this so-called stagnation of MMR in Sri Lanka [2].

### Postpartum family planning

Many women wants to delay the next pregnancy after a childbirth, but are unclear about postpartum family planning (PPFP) methods in most of the instance [3]. During the first year postpartum, 40% of women are estimated to have an unmet need for contraception [4]. Closely spaced pregnancies give rise to adverse maternal, perinatal and infant outcomes [5]. The PPIUD enables women to leave the birth facility with a safe and extremely effective LARC ready in place [6]. Aimed to study the institutionalization of PPIUD services at Teaching Hospital, Mahamodara, Galle (THMG) and to study the feasibility, challenges and barriers for implementation.

### Method

#### Study design and setting

A retrospective observational study was carried out regarding the details of the PPIUD Project from 2014 to 2019 in THMG.

#### Funding, advocacy and stakeholders

This PPIUD project was carried out by the Sri Lanka College of Obstetricians and Gynecologists (SLCOG) with the collaboration of Family Health Bureau (FHB), Ministry of Health, Sri Lanka as the key stakeholder. The project had been funded by International Federation of Gynaecology and Obstetrics (FIGO).

#### Basic outline of the project in Sri Lanka

**Fig. 1:**
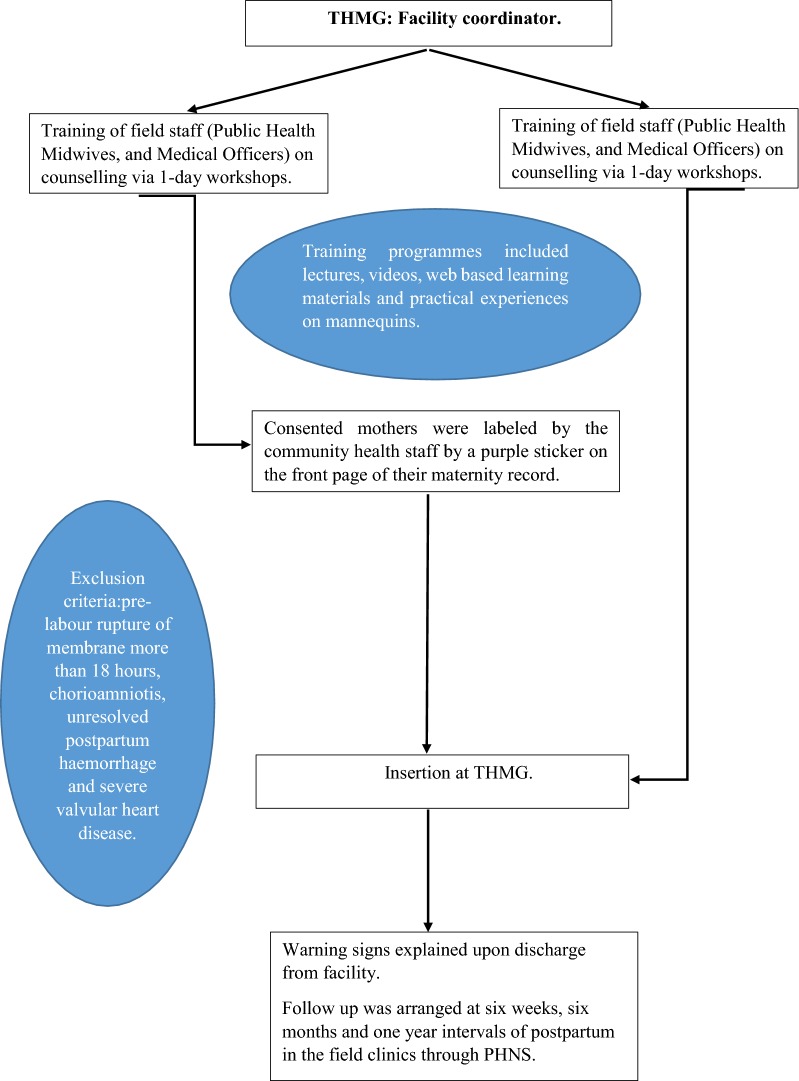
Basic outline of the project in Sri Lanka. *THMG* Teaching Hospital Mahamodara Galle, *PHNS* Public Health Nursing Sister

This has outlined in Fig. 1. The PPIUD project was implemented in two phases: phase I, involving six hospitals began in 2013; and phase II with 12 hospitals began in 2015 [7]. Hospitals were selected based on the number of deliveries and other criteria covering implementation issues. THMG was included in phase I. A facility coordinator was appointed to conduct the project at THMG (Author LD) by the SLCOG. Trainer’s Notebook on Postpartum Intrauterine Contraceptive Device (PPIUD) Services provided the necessary information [8].

#### Data collection

A trained data collecting officer was involved in PPIUD project at THMG. Women were interviewed at four points: baseline, 4–8 weeks postpartum, 6 months postpartum and 1 year postpartum. Outcome measures were assessed quantitatively using an interviewer-administrated questionnaire. The regular review of the data set was performed by the facility coordinator to ensure the quality and accuracy of data.

Details regarding the percentage of community staff trained for counseling and follow up, percentage of clinicians trained for PPIUD insertion, percentage of antenatal women counseled for PPFP percentage of antenatal women consented for PPIUD, percentage of women with withdrawal of the consent, percentage of women who had spontaneous expulsion, percentage of women who got pregnant at the end of 1 year and sustainability; continuation of PPIUD at the end 1 year were taken in addition to the basic demographic data. Data was collected using an electronic assisted personal interview (EAPI) format using commcare survey management system on android based tablets.

#### Data management and analysis

Data had been securely transferred from the android tablet on to a commcare supported secure cloud survey provided by the FIGO. Nominal and categorical variables were presented as percentages.

#### Ethical considerations

Ethical approval was obtained from the Ethical Review Committee, Faculty of Medicine, University of Ruhuna. Prior approval was also taken from SLCOG to publish the data taken from THMG separately.

### Results

#### Basic demographic characteristics

Total of 46,815 deliveries had occurred in the facility during the study period. Out of that 23,117 (49.4%) women had been counseled. Out of these counselled women 5775 (25.0%) of them were primigravida and 892 (3.9%) were teenage mothers. Total of 14,051 (60.8%) women were interviewed, but only 772 (5.5%) women consented for PPIUD. Consent withdrawal was seen in 29 (3.8%) cases.

#### Training

A total of 409 community health staff (MOH, PHNS, and PHM) were trained for counseling and follow up. Community staff participation for the training sessions were highly satisfactory. In hospital setting, 117 clinicians were trained for insertion. THMG is a tertiary care setting where a lot of doctors are having 6 months to 1 year rotational appointments leading to a high number of trained clinicians (n = 117) compared to PPIUD uptake. Loss to attendance for follow up was a major problem identified. Therefore it was decided to send reminders for postnatal mothers to attend for follow up. During the project five clinicians were identified having high failure rates with improper PPIUD placement. They were trained again to acquire the expected level of skill competency. PPIUD was removed after insertion in 36 (7.7%) cases before discharging from the birth facility.

#### PPIUD Uptake

PPIUD uptake (out of interviewed women) had been occurred in 470 (3.4%) cases. Out of these 470, 161 (34%) insertions were intra-caesarean. Out of 302 (39.1%) who did not give the consent for PPIUD only 50 women have given the reasons for non-insertion. Main reason for non-insertion of planned PPIUD was contraindications. Table 1 summarizes common reasons for non-insertion of a planned PPIUD.

**Table 1:** Common reasons for non-insertion of a planned PPIUD

Reasons for non-insertion of PPIUD. (Total non-insertions = 50)	n (%)
Contraindications	20 (66.7)
Mother has changed her mind	1 (2.0)
Technical problems at the time of insertion	8 (16.0)
Unavailability of IUD at the time of insertion	1 (2.0)
IUD Removed	5 (10.0)
IUD expelled with blood	6 (12.0)
Consent given for sterilization	1 (2.0)
Purple sticker was missing	1 (2.0)
Consent given for sterilization	1 (2.0)
Stillbirth	1 (2.0)
Other	8 (16.0)

*PPIUD* Postpartum intrauterine device, *IUD* Intrauterine device

#### Follow up

One year follow up data was available from 119 cohort of women with PPIUD. The total number of PPIUD related complications occurred in 8 (1.7%) cases. Table 2 shows the outcomes of follow up visits of all PPIUD insertions. There were no cases of perforation or any unexpected pregnancy. Symptoms of abnormal vaginal discharge with pelvic infection were reported by three (2.5%) women during follow up. Spontaneous expulsion had occurred in eight (6.7%) cases. PPIUD removal was done prematurely on request for medical or personal reasons in eight (6.7%) cases.

**Table 2:** Outcome of PPIUD at follow up visits

	Frequency (n = 119)	%
PPIUD in situ (uncomplicated) complications	95	79.8
(i) Perforation	0	0
(ii) Abdominal pain	1	0.8
(iii) Infection(vaginal/pelvic)	3	2.5
(iv) Irregular bleeding	4	3.4
Efficacy		
(i) Pregnancy	0	0
(ii) Expulsion	8	6.7
(iii) Discontinuation	8	6.7

*PPIUD* Postpartum intrauterine device

### Discussion

This SLCOG-led PPIUD project in collaboration with FIGO has resulted in worthwhile efforts to provide the benefits of this LARC within the birth facility itself in Sri Lankan setting. PPIUD uptake (out of interviewed women) was 3.4% and this is comparable with national figures in Sri Lanka [8]. However, rate of counseling was almost half of the women who delivered at the THMG and PPIUD had not been inserted in 39.1% of consented women due to various reasons as in Table 2. Implementation of safe, effective and sustained PPIUD services at a clinical setting is a complex process which involved informed demand, infrastructure, supplies, personals, coordination of care and management system. The current study shows the basic outline that provision of PPUD services are safe and effective option of PPFP and feasible to implement at THMG.

Amongst the women studied at follow-up, there were no cases of uterine perforation. No previous studies have reported uterine perforation after insertion [9]. Majority (79.8%) had uncomplicated follow up in the present study. Expulsions were seen in eight (6.7%) cases. Several regional studies using similar technique and timing with trained clinicians have shown comparable expulsion rates and findings [9, 10]. With regards to the safety of PPIUD in terms of infection, conception and perforation, only three cases of infection (2.5%) were seen in the current study and others have reported 1.75% [9] and 0.84% [10]. In total of 36,766 women have had PPIUD inserted across the 6 countries and follow up of just over 50% demonstrated that expulsion rates were lower than previously reported at 2.6%. Rates of severe infection has low as 0.01% and there was no single case of perforation [11]. In the present study, 1 year follow up rate was 25% and this is lower than the global findings [11]. However expulsion rate in the study was 1.7% which is well below the reported rate from six countries mentioned [11]. In present study, no cases of failure in the form of pregnancy was observed. Similar findings reported in two studies [12, 13]. Extremely low rate of PPIUD associated complications such as perforation, infection, bleeding and pain, indicates the safety of the PPIUD.

With regards to initial recruitment, women need reliable unbiased information about PPIUD to make an informed choice. Basically implementation of this PPIUD mainly needs addressing women properly and maintaining a continuous follow up with minimal need of additional infrastructure. Minimal need of infrastructure facilities further strengthens the PPIUD as an appropriate method for PPFP for low-middle income countries (LMIC). Specially addressing myths and taboos about PPIUD is a timely need. Development of printed materials videos on this in local language can be useful for the community and antenatal education giving a potential impact. Overall coordination of the services are of paramount importance for the uninterrupted and smooth function of the PPIUD service. This involves supervision of community and hospital staff, arrangement of training sessions, maintenance of necessary records, communication and sharing of information as well as communication with the central level. In this study the facility coordinator was appointed by the SLCOG (author LD) with the above responsibilities and the coordinator had regular discussions and review meetings with both community and hospital staffs. The study revealed that the only half of antenatal women received PPFP counseling and two-thirds of those were interviewed regarding PPIUD. Too overcome this deficiency, effective awareness programmes are essential component of PPFP services to enhance education on PPIUD. This can be achieved with media advertisement, distribution of information brochures and health education programmes especially addressing mythos and taboos as mentioned in above. Contributing factors for less uptake could be due to religious movement against contraception, attitudes of obstetricians towards PPIUD and ethnic diversity in the setting. All of these are reversible factors for better maternal and child health outcomes and can make an obvious difference in the foreseeable future.

Another study revealed that women undergoing caesarean deliveries with greater probability of accepting the PPIUD [9]. In our study vaginal deliveries had a majority of insertions. Although all the women who received immediate PPIUD were advised to come for a follow-up, only a few (25.3%) were turned up. Indian study also had a comparable follow up rate (28.8%) indicating parallel socio-economic levels in low-middle income countries [9]. Indian study has shown that follow up of PPIUD was 65.2% and they have contacted through telephone offering transportation incentives [10]. However, Shukla et al. reported a follow-up of 78.7% in a 5-year prospective longitudinal study [14]. Implementation with further capacity building of the existing as well as new team members and appointing master trainers nationwide can increase the uptake. Involvement of the SLCOG and FHB demonstrate sustainability of the PPIUD service in Sri Lanka.

### Conclusions and recommendations

This short report gives the impression that PPIUD can be successfully implemented in LMIC settings and also provides a feedback for the policy makers to take the necessary actions to improve the uptake of this cost effective, safe PPFP method. Low uptake has to be addressed. Development of PPIUD service allows more women to access PPFP. This prevents unintended pregnancies, and extend inter-pregnancy intervals. Through ongoing research and development of a shared-learning culture, it is possible to bring the PPIUD service to a more widely available level across Sri Lanka as well as other countries.

## Limitations

Additional data at follow up could have been obtained to analyze to see any significant difference between age groups, parity and vaginal versus intra-caesarean insertions. It would be more valuable if any survey had been conducted among the community staff to see any potential barriers in implementation at the grass root level.

## Data Availability

Datasets generated from this study will be available from the corresponding author (MP) upon a reasonable request.

## Abbreviations

PPFP: Postpartum family planning
WHO: World Health Organisation
LARC: Long acting reversible contraceptives
IUD: Intra uterine device
MMR: Maternal mortality ratio
THMG: Teaching hospital, mahamodara, galle
SLCOG: Sri Lanka college of obstetricians and gynecologists
FHB: Family health bureau
FIGO: International federation of gynaecology and obstetrics
LMIC: Low middle income countries
MOH: Medical officer of health
PHM: Public health midwife
PHNS: Public health nursing sister
